# Biogeography, phylogeny, and morphological evolution of central Texas cave and spring salamanders

**DOI:** 10.1186/1471-2148-13-201

**Published:** 2013-09-17

**Authors:** Nathan F Bendik, Jesse M Meik, Andrew G Gluesenkamp, Corey E Roelke, Paul T Chippindale

**Affiliations:** 1 Department of Biology, University of Texas at Arlington, Arlington, Texas 76019, USA; 2 City of Austin, Watershed Protection Department, Austin, Texas 78704, USA; 3 Department of Biological Sciences, Tarleton State University, Stephenville, Texas 76402, USA; 4 Texas Parks and Wildlife Department, Austin, Texas 78744, USA

**Keywords:** *Eurycea*, *Blepsimolge*, Salamanders, Troglobites, Cave adaptation, Morphological evolution, Troglomorphism

## Abstract

**Background:**

Subterranean faunal radiations can result in complex patterns of morphological divergence involving both convergent or parallel phenotypic evolution and cryptic species diversity. Salamanders of the genus *Eurycea* in central Texas provide a particularly challenging example with respect to phylogeny reconstruction, biogeography and taxonomy. These predominantly aquatic species inhabit karst limestone aquifers and spring outflows, and exhibit a wide range of morphological and genetic variation. We extensively sampled spring and cave populations of six *Eurycea* species within this group (eastern *Blepsimolge* clade), to reconstruct their phylogenetic and biogeographic history using mtDNA and examine patterns and origins of cave- and surface-associated morphological variation.

**Results:**

Genetic divergence is generally low, and many populations share ancestral haplotypes and/or show evidence of introgression. This pattern likely indicates a recent radiation coupled with a complex history of intermittent connections within the aquatic karst system. Cave populations that exhibit the most extreme troglobitic morphologies show no or very low divergence from surface populations and are geographically interspersed among them, suggesting multiple instances of rapid, parallel phenotypic evolution. Morphological variation is diffuse among cave populations; this is in contrast to surface populations, which form a tight cluster in morphospace. Unexpectedly, our analyses reveal two distinct and previously unrecognized morphological groups encompassing multiple species that are not correlated with spring or cave habitat, phylogeny or geography, and may be due to developmental plasticity.

**Conclusions:**

The evolutionary history of this group of spring- and cave-dwelling salamanders reflects patterns of intermittent isolation and gene flow influenced by complex hydrogeologic dynamics that are characteristic of karst regions. Shallow genetic divergences among several species, evidence of genetic exchange, and nested relationships across morphologically disparate cave and spring forms suggests that cave invasion was recent and many troglobitic morphologies arose independently. These patterns are consistent with an adaptive-shift hypothesis of divergence, which has been proposed to explain diversification in other karst fauna. While cave and surface forms often do not appear to be genetically isolated, morphological diversity within and among populations may be maintained by developmental plasticity, selection, or a combination thereof.

## Background

Radiations of karst limestone fauna are characterized by multiple invasions into cave systems that may result in convergent or parallel phenotypic evolution [1-4], genetically admixed populations [5], and alternating periods of isolation and gene flow between surface and cave populations [6]. These complex histories pose problems for phylogenetic and phylogeographic reconstruction, assessment of biodiversity [7], and inference of evolutionary transitions [8]. Because of the discontinuous and sensitive habitats that define karst systems, many are “hotspots” of threatened and endangered species. The Edwards Plateau of central Texas exemplifies this pattern and harbors diverse, endemic invertebrate and vertebrate species [9]. The Plateau is an uplifted Cretaceous limestone that has eroded to form a complex, highly subdivided aquifer system with numerous springs and water-filled caves. These karst habitats have been widely colonized by a group of lungless, primarily paedomorphic (retaining aquatic larval form while attaining reproductive maturity) spelerpine plethodontid salamanders of the genus *Eurycea*[10-14], of which thirteen species are recognized. Given that many populations of *Eurycea* in Texas are threatened by effects of urbanization, such as declining water quality and decreased water levels from pumping of the Edwards and Trinity aquifers [15,16], a detailed understanding of genetic structure and diversity in the group is essential (particularly with regard to identification of species and their distributions). In addition, these salamanders exhibit extensive morphological variation associated with both cave (subterranean) and surface habitats, making them well suited for investigation of parallel evolution of morphological traits in similar environments (e.g., [17]).

The central Texas *Eurycea* have a complicated taxonomic history [10,11,14,18-21], in part because convergence or parallelism in cave populations has confounded studies that relied solely on morphology or morphometrics (e.g., [22], but see [17]). Conversely, morphological conservatism (primarily among surface-dwellers) has also led to underestimation of species diversity [10,20]. Where morphological data have failed, molecular phylogenetic studies have clarified higher-level, and in some cases species-level relationships within the group [10,21]. Although members of this group belong to the genus *Eurycea* under a traditional Linnaean classification scheme, Hillis et al. [21] recognized additional well-supported clades under an unranked system (PhyloCode [23]). The deepest split (at least 15 Ma BP; [24]) corresponds to a clade occurring north of the Colorado River (*Septentriomolge*) and a clade south of the Colorado River (*Notiomolge*, consisting of clades *Blepsimolge* and *Typhlomolge*). The distribution of *Blepsimolge* includes caves and springs from the vicinity of Austin and San Marcos in the east and extending west to Val Verde County. *Typhlomolge* comprises exclusively subterranean species sister to and essentially sympatric with *Blepsimolge* along the southeastern periphery of the Edwards Plateau [10,21]. *Blepsimolge* can further be divided into eastern and western groups, which appear to be geographically discontinuous and are well differentiated genetically [10,20]. The western group is often termed the *E. troglodytes* complex [10,20]. Here we focus on the clade *Blepsimolge* from the eastern region, which comprises populations assigned to six nominal species (*E. latitans*, *E. pterophila*, *E. nana*, *E. neotenes*, *E. sosorum* and *E. tridentifera*). Hereafter, we refer to these populations as the “eastern *Blepsimolge*”. Relationships among many populations remain uncertain and the validity of some species in this group is questionable [10,20].

Populations of central Texas *Eurycea* exhibit habitat-associated morphological variation. Surface populations, found in springs, spring outflows and low-order streams, are typically pigmented, with muscular limbs, elongated trunks, and well-developed eyes. Subterranean populations exhibit a continuum of variation ranging from surface-like to highly troglomorphic [22], with the most extreme examples being *E. tridentifera* (eastern *Blepsimolge*) and, at the furthest end of the “troglomorphic spectrum”, *E. rathbuni*, *E. robusta*, and *E. waterlooensis* (*Typhlomolge*). These “extreme” species have vestigial eyes, long spindly limbs, shortened trunks, broad flattened snouts, and highly reduced skin pigmentation. Although morphological convergence between *E. tridentifera* and *Typhlomolge* has been established [17], the extent of morphological divergence (and parallelism) among other surface and subterranean populations of the eastern *Blepsimolge* has not been formally evaluated. Patterns of morphological variation within and among these populations are complex, as there may have been multiple independent invasions into cave systems, and because of the structural and hydrogeological complexity of cave and surface habitats. Here we provide a phylogenetic analysis of the eastern *Blepsimolge* based on mtDNA sequence data from extensive sampling of surface and subterranean sites, and we relate our phylogenetic hypothesis to new data on morphological variation within the group. Specifically, we characterize broad patterns of morphometric variation using multivariate analysis, evaluate the extent and phylogenetic distribution of cave-associated morphologies, and discuss evolutionary, developmental, and taxonomic implications.

## Methods

### Taxon sampling for molecular analyses

Salamanders were collected from springs and caves across a seven county area of the southeastern Edwards Plateau. Our data set includes more than triple the number of sites sampled in previous studies [10,21] including extensive, fine-scale examination of critical, and formerly unsampled and/or previously inaccessible regions. Data were obtained for 112 specimens collected from 45 springs and 26 caves in the southeastern Plateau region. These include representatives of the *E. latitans* complex (sensu [10,20])*, E. pterophila, E. nana, E. neotenes, E. tridentifera* and *E. sosorum* (eastern *Blepsimolge*), plus multiple newly sampled populations not previously assigned to species and outgroup samples from *E. rathbuni* (*Typhlomolge*) and the *E. troglodytes* complex (western *Blepsimolge*). We adhered to animal welfare protocols outlined by the University of Texas Arlington (IACUC # A.07.021). Specimen details are available in Additional file 1.

### Laboratory methods

DNA was extracted from muscle or liver tissue using several methods. For all of the tissue samples obtained between 2003 and 2007, DNA was extracted using the DNeasy kit from Qiagen. DNA for specimens collected prior to 2003 was extracted primarily using the STE method described by Hillis et al. [25] and a modification of the Chelex extraction method [26], described by Chippindale et al. [10]. We focused on two mitochondrial DNA (mtNDA) sequence regions for our phylogenetic analysis: a 1110 bp fragment of cytochrome *b* (Cytb), and a 619 bp fragment of NADH dehydrogenase subunit 2 plus adjacent tRNA^TRP^ and partial tRNA^ALA^ (ND2). Most PCR products were amplified using a standard *Taq* polymerase (New England Biolabs or Promega) or Hot Start *Ex-Taq* (Takara-Mirus) on MJ Research PTC 200 gradient and PTC 100 thermal cyclers. Amplification for PCR and sequencing was performed using the primers listed in Table 1. PCR conditions that yielded the most consistent results were as follows: Reactions consisted of 1–2 μl of dilute DNA (typically 10-50 ng of DNA, but sometimes as high as 300 ng), 0.5–1.0 μM of each primer, 0.75 mM dNTPs mix, polymerase buffer (1.5 mM MgCL_2_), and 1–2 U *Taq* (or 0.5 U *ExTaq*) polymerase in a total volume of 20 μl. Occasionally, 2.5% DMSO (final concentration) was used in difficult PCR reactions. Thermal cycling conditions varied greatly depending on the template and difficulty of amplification. Typical conditions were as follows: Step 1: 96° 3 min; Step 2: Annealing temp. 50° 30 s; Step 3: 72° 1 min/kb; Step 4: 96° 20 s; Step 5: repeat steps 2–4 (× 30); Step 6: 72° 10 min; Step 7: 4° hold. Variations of this profile included a step-up annealing temperature, whereby the first 2 or 3 replications included a 3–5° lower annealing temperature, and then were raised to the standard annealing temperature for the remaining cycles. Some PCR reactions (especially for difficult samples) were performed using Phusion or Phusion II DNA polymerase (New England Biolabs), generally following manufacturer’s instructions but in 5–10 μL total volumes and including BSA and DMSO (“1X” and 3.0 mM final concentrations, respectively). Often, these reactions were performed “semi-nested” with a third primer added at 0.1X the concentration of each of the other two (PGLU-TAT for Cytb, and cytochrome oxidase I primer MVZ 202 for ND2; Table 1). Typical reaction profiles involved initial denaturation for 2 min at 98°, subsequent denaturations at 98° for 10 s, annealing times of 5–10 s, and extension times of 10–20 s at 72° with a final 10 min extension step. Generally “touchdown” methods were used in which annealing temperatures were successively dropped in increments of 2-3°, from about 58–50° for Cytb and 62–55° for ND2, with increasing numbers of cycles at each lower temperature for a total of 35–40 cycles.

**Table 1:** List of primers used to amplify and sequence gene fragments in this study

**Primer name**	**Primer sequence (5’-3’)**	**Amplified region**
PGLU	GAARAAYCANTRTTGTATTCAAC	Cytb
PGLU-TAT**	GAARAAYCANTRTTGTATTCAACTAT	Cytb
MVZ-15 [27]	GAACTAATGGCCCACACWWTACGNAA	Cytb
HEM-CB1-5’	CCATCCAACATCTCAGCATGATGAAA	Cytb
CYTBTN5Fv2	CATATTTAGGRGAAACACTTGTTCA	Cytb
CYTBTYPHmR	GTCKGGGYTAGAATTAATTCCTG	Cytb
EurTXCRThr	GYCAATGTTTTTCTAAACTACAACAGCATC	Cytb
METf L4437	AAGCTTTTGGGCCCATACC	ND2
COIr H5934	TGCCAATATCTTTGTGATTTGTT	ND2
ND2f L5002*	AATCAACCACAAATCCGAAAAAT	ND2
ASNr H5692*	TTAGGTATTTAGCTGTTAA	ND2
MVZ 202** [28]	GCGTCWGGGTARTCTGAATATCGTCG	ND2

ND2 includes adjacent Ala and Trp tRNA genes.

*indicates primer was only used for sequencing, and not PCR.

** indicates external primer for nested PCR.

PCR products were purified using Qiagen gel extraction or PCR purification kits following the manufacturer’s protocols, or if nonspecific products were absent, a combination of exonuclease I and shrimp alkaline phosphatase enzymes (USB) were used to digest single-stranded DNA and phosphorylate dNTPs. Both strands of each amplicon were sequenced for complete or nearly-complete overlap for most templates using ABI Big Dye v3.1 chemistry. Unincorporated products were removed via ethanol precipitation using 0.75 M sodium acetate and 125 mM EDTA. Applied Biosystems 377 and 3130xl machines were used for sequencing.

### Alignment and phylogenetic analysis

Raw sequence chromatograms were edited with Sequencher v4.2, v4.3 and v4.5 (Gene Codes Corp., Ann Arbor, MI, USA). Multiple alignments were conducted with MEGA v5 [29] using MUSCLE [30]. For phylogenetic analyses, *Eurycea rathbuni* was chosen as the outgroup because it is well supported as sister to *Blepsimolge* in previous molecular studies [10,17,21]. Bayesian phylogenetic analysis of the combined Cytb and ND2 gene segments was run using MrBayes v3.1.2 [31] on the CIPRES Science Gateway [32]. Nucleotide models of evolution were determined using jModeltest v0.1.1 [33,34]. In MrBayes, the parameters statefreq, revmat, shape and pinvar were allowed to vary by gene segment. Default priors set by MrBayes were used except for the branch-length priors; branch-length priors were set as exponential with means of 1, 0.1, 0.01 and 0.001 in four separate runs to test prior sensitivity, since a large branch-length prior can result in unrealistically long trees [35]. Each analysis was run twice with 4 chains (one cold, three heated), 5 million MCMC generations and a sample frequency of 100. Burn-in was determined by examining the log files generated by MrBayes using program Tracer v1.5.0 [36]; parameter traces were visually assessed for stationarity. The post-burn-in trees from both runs were combined to calculate a majority-rule consensus with a cutoff of 50%.

### Morphological data and analyses

We measured a series of ten standardized distances based on external morphology from 255 ethanol-preserved specimens catalogued at the following collections: Texas Natural History Collection, The University of Texas at Austin; Museum of Vertebrate Zoology, University of California, Berkeley; and the Amphibian and Reptile Diversity Research Center at the University of Texas at Arlington [Additional file 2]. Our sample was primarily organized by collecting locality and included representatives of each species of eastern *Blepsimolge*, as well as comparative material from the exclusively subterranean species of *Typhlomolge* (*E. rathbuni* and *E. waterlooensis*). Measurements ≥ 20 mm were taken with a digital caliper and rounded to the nearest 0.1 mm; measurements < 20 mm were made with an ocular micrometer mounted on a dissecting microscope and rounded to the nearest 0.01 mm. Morphometric variables were selected from Chippindale et al. [10] and included the following measurements: AG (axilla-groin length); ALL (anterior limb length, from insertion to tip of third toe); ED (eye diameter); HLA (head length A, distance from tip of snout to gular fold); HLB (head length B, distance from posterior margin of eye to posterior-most gill insertion); HLC (head length C, distance from tip of snout to posterior-most gill insertion); HLL (hind limb length, from insertion to tip of third toe); HW (head width at rictus of mouth); IOD (interocular distance); SL (standard length, distance from tip of snout to posterior margin of vent). Eye diameter was determined using maximum ocular disc diameter, i.e., portion of dark pigment including and surrounding the focusing portion of the eye, under 64X magnification with backlighting. Results were highly consistent and repeatable among specimens examined by PC, CR, and AG. We used principal components analysis (PCA) to characterize broad patterns of morphological variation, and to explore correlation structure among variables. PCA was carried out with Systat 12.02 (Systat Software, Inc., Chicago, IL, USA), using the correlation matrix derived from log_10_-transformed variables. Although greater separation of groups in ordination space could be achieved by reducing the influence of size through analysis of measurement residuals, we elected against this procedure for the following reasons: (1) the joint influence of size and shape on phenotype is biologically relevant (e.g., [37]); (2) in our data set, individual measurements scaled to SL with different slopes and functions, making residuals problematic to compare with each other [38]; and (3) measurements spanned almost three orders of magnitude; thus, a common scaling factor would likely model more noise than signal for shorter measurements. Furthermore, variance attributable to size and shape cannot be separated with only linear measurements [39]. Some of these issues also influence raw or transformed measurement data that is input into PCA, but may be compounded by further processing.

## Results

### Phylogenetics

The length of the combined Cytb and ND2 alignment was 1729 bp. Thirty-four sequences were slightly shorter than the others; unsequenced regions were treated as missing data. The maximum number of missing bases (including both Cytb and ND2) was 91 for one specimen; the average value was 7. Including outgroup taxa, 380 sites were variable and 226 of these were parsimony-informative. Of 107 eastern *Blepsimolge* specimens, 47 haplotypes were recovered from 71 different springs and caves, for which 155 sites were variable and 72 were parsimony-informative. Genbank accession numbers are KC355860–KC355971 and KC355972–KC356083 for Cytb and ND2, respectively [see Additional file 1]. The model with the highest AICc score according to jModeltest was GTR + G for both Cytb and ND2 alignments. Branch length priors of exponential mean 1, 0.1 and 0.01 resulted in unrealistically long tree lengths; results from the smallest (exponential mean of 0.001) branch-length prior analysis are presented (mean tree length = 0.28, LCL = 0.26, UCL = 0.30), although there were no significant differences in tree topologies. Samples from the first 500,000 iterations were discarded as burn-in.

**Figure 1:**
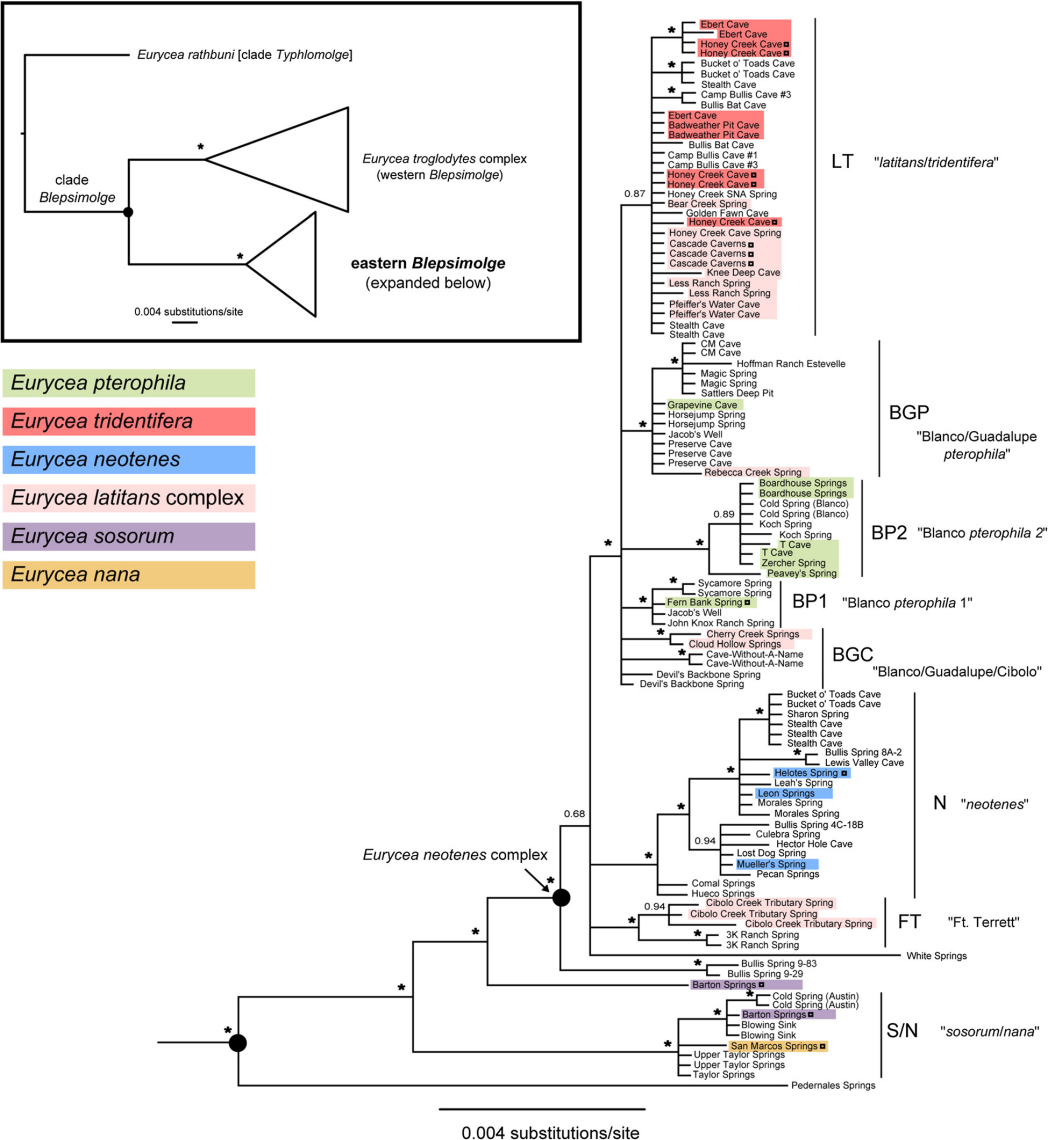
**Fifty percent majority-rule consensus phylogram of eastern *Blepsimolge *based on Bayesian analysis.** Posterior probabilities of node support greater than or equal to 95% are indicated by asterisks. Species designations (indicated by colored blocks) follow those given by [10], and we include all of the same populations from their molecular analysis plus numerous new samples. Hollow squares indicate topotypical specimens.

**Figure 2:**
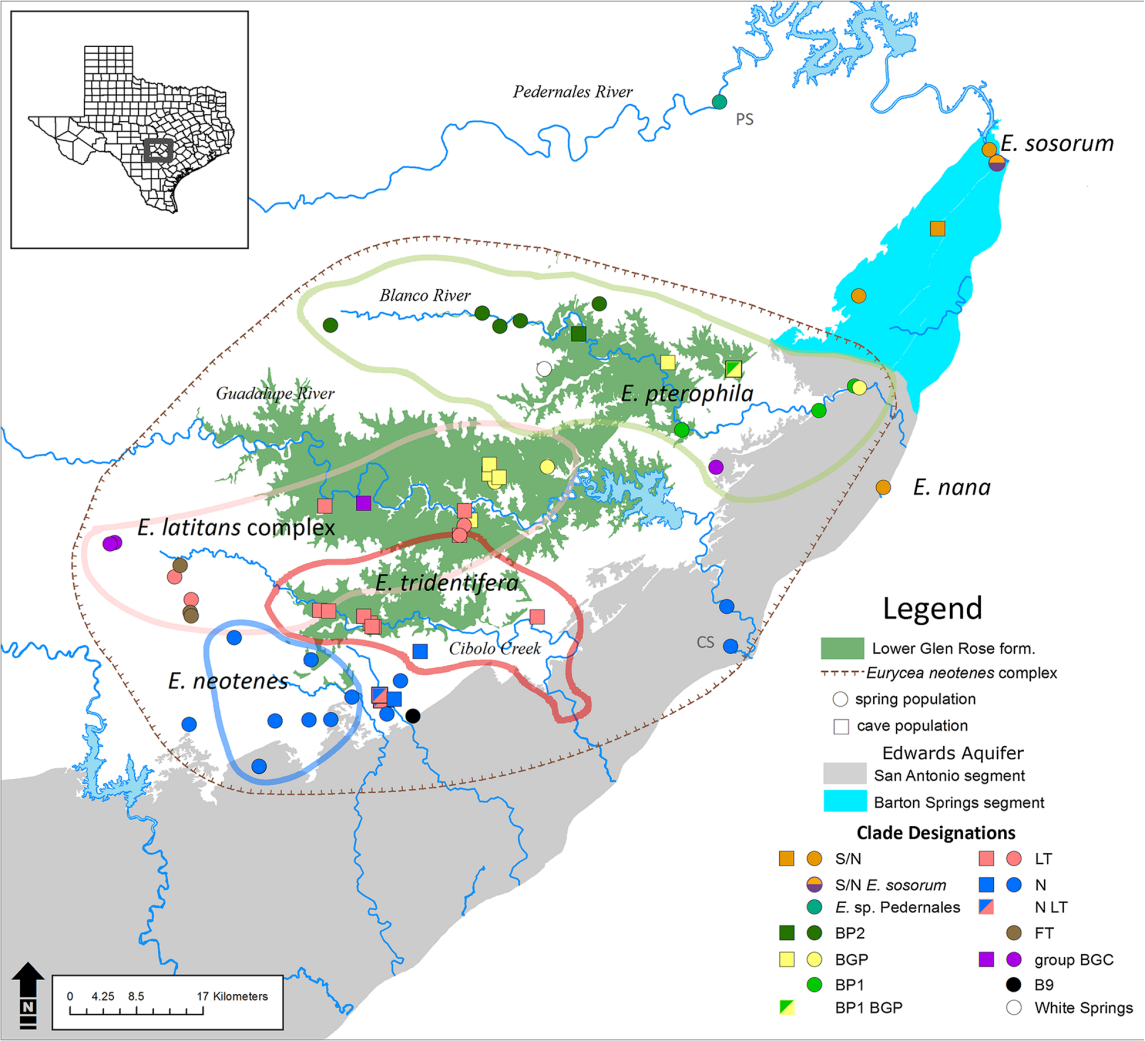
**Geographic distribution of eastern *Blepsimolge *mtDNA clades in relation to species boundaries, habitat and major physiographic features.** Squares and circles represent spring and cave localities, respectively. Clade associations are labeled by color for each sampled population; populations with haplotypes from two distinct clades are bicolored. Approximate distributions for *Eurycea neotenes* complex species (colored lines) are drawn according to designations by [10] although these designations are not entirely consistent with our phylogenetic hypotheses. Similarly, physiographic boundaries also appear to be poor predictors of mtDNA clade distributions. B9 = Bullis springs 9–83 and 9–29; CS = Comal Springs; PS = Pedernales Springs.

Phylogenetic results agree with those of previous studies regarding the deepest splits within *Eurycea* from the southern Edwards Plateau (exclusive of clade *Typhlomolge*, the outgroup), and the deep nodes are strongly supported. The most basal split is between the *E. troglodytes* complex (western *Blepsimolge*) and the eastern *Blepsimolge*[10,21]). Also consistent is the distinctiveness of a population that may represent an undescribed species, from Pedernales Springs [10]. *Eurycea* sp. Pedernales is sister to the remaining eastern *Blepsimolge*, followed by *E. nana* from the type locality, San Marcos Springs (and individuals that appear to represent *E. sosorum* but possess *E. nana*-like haplotypes; collectively clade S/N) (Figure 1). *Eurycea sosorum* (primary haplotype at Barton Springs, the type locality) is sister to the remaining clade, which includes populations representing *E. latitans*, *E. neotenes*, *E. pterophila* and *E. tridentifera* plus others that have not been assigned to species (Figure 1). We refer to this clade as the *Eurycea neotenes* complex (after the first-described member of the group [40]; see Discussion), which is distributed throughout the Cibolo, Guadalupe and Blanco river watersheds (Trinity Aquifer), but also includes several populations that occur along the southeastern edge of the Edwards Plateau (Edwards Aquifer; Figure 2).

**Figure 3:**
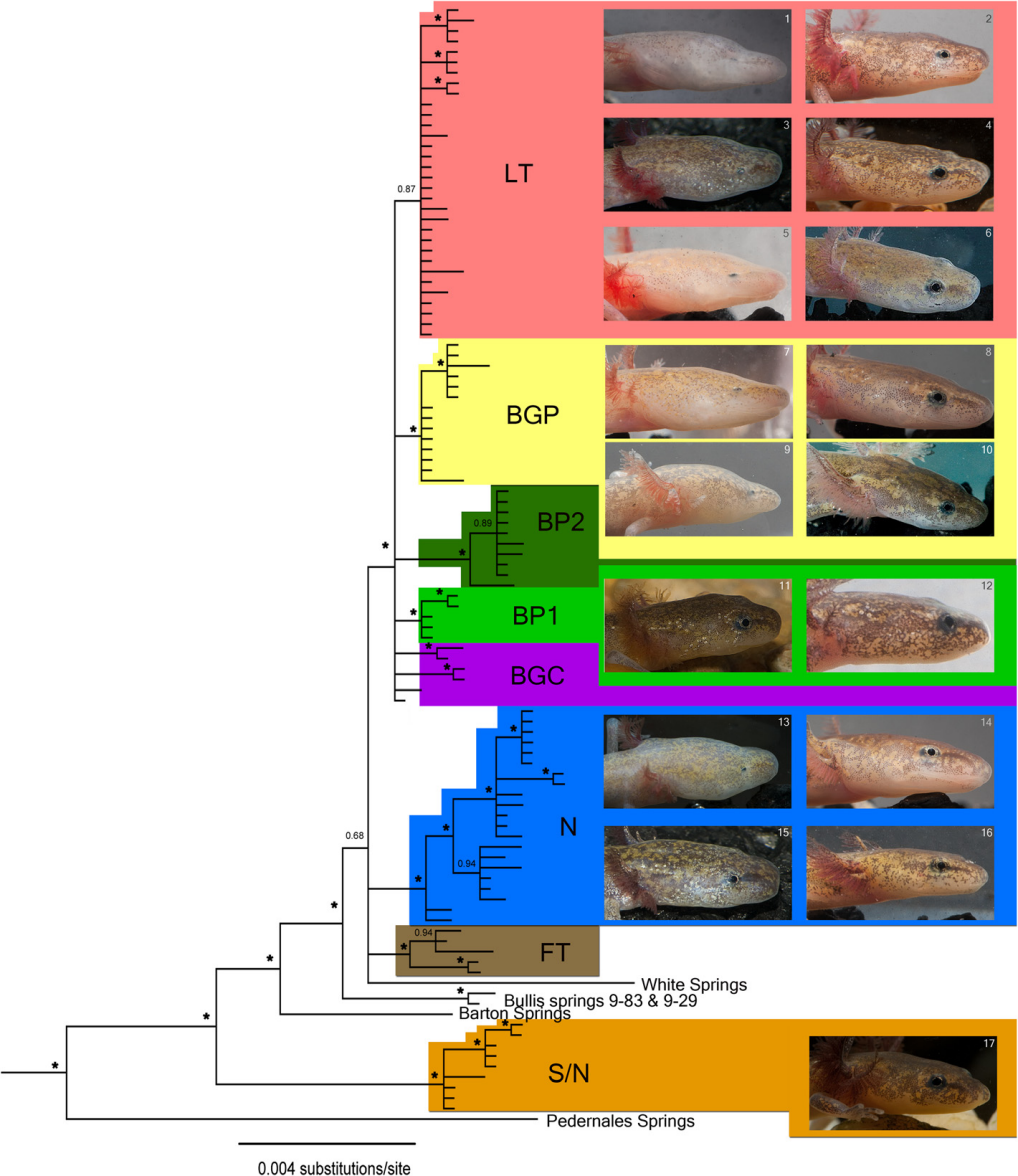
**Diversity of head morphology and pigmentation within the eastern *Blepsimolge*.** Parallel patterns of morphological evolution are evident in the troglomorphic specimens from clades LT, N and BGP, although all labeled clades contain surface forms (i.e., having fully-developed eyes and dark pigmentation). Localities for individuals pictured are as follows: **1** Honey Creek Cave, **2** Cascade Caverns, **3** Camp Bullis Cave #3, **4** Cascade Caverns, **5** Bullis Bat Cave, **6** Golden Fawn Cave, **7** Preserve Cave, **8** CM Cave, **9** Preserve Cave, **10** Hoffman Ranch Estavelle, **11** Fern Bank Spring, **12** Jacob’s Well, **13** Hector Hole, **14** Lewis Valley Cave, **15** Sharon Spring, **16** Morales Spring, **17** Taylor Springs.

Although there are some highly supported clades within the *E. neotenes* complex, primarily comprising geographically proximate populations, these do not strictly correspond to currently recognized species boundaries (Figures 1 and 2; [10]). Divergences among populations within this region are low (average uncorrected p-distance = 0.4%) and there is extensive inter- and sometimes intrapopulation morphological variation (e.g., Figure 3). The weakly supported clade LT includes representatives (including topotypes) of both *E. latitans* and *E. tridentifera* (Figure 1). Clades BP1, BP2 (Blanco River drainage populations of *E. pterophila*) and BGP (Blanco & Guadalupe River *E. pterophila*) each contain populations assigned to *E. pterophila*[10], but form a polytomy in the 50% majority-rule tree with clade LT and several other populations (Figure 1). Group BGC contains four populations distributed across the Blanco, Guadalupe and Cibolo drainages that form a polytomy with BP1, BP2, BGP and LT. Collectively BP1, BP2, BGP, LT and BGC form a highly supported clade that includes most populations previously assigned to *E. latitans*, *E. tridentifera* and *E. pterophila*. Clade N includes populations assigned to *E. neotenes* as well as the population from Comal Springs (Figure 1), which has been suggested to be a distinct species [10]. Clade FT includes populations previously assigned to *E. latitans*[10] that occur in or near the Fort Terrett limestone formation. Clades N, FT and the recently discovered population at White Springs form a polytomy, while the previously unsampled Bullis Springs 9–29 and 9–83 are weakly supported as sister to the rest of the *E. neotenes* complex. The latter three populations exhibit relatively high genetic divergences compared to the rest of the *Eurycea neotenes* complex [average uncorrected p-distances 1.2% (White Springs) and 0.7% (Bullis Springs 9–29 & 9–83)].

Patterns suggestive of mitochondrial introgression are also evident for several populations (Figure 1). Haplotypes from Jacob’s Well (Blanco River drainage) occur in clades BP1 and BGP. Additionally, there is potential introgression and/or gene flow between adjacent populations of clades LT and N, as haplotypes from both clades are found in the Stealth Cave and Bucket o’ Toads Cave populations. Our sample of *E. sosorum* (putatively endemic to Barton Springs) also contains two distinct mitochondrial haplotypes, one unique to Barton Springs at high frequency (approximately 70%; unpublished data, [41]) and one that groups with that of *E. nana* (San Marcos Springs) and other Barton Springs segment populations (Blowing Sink, Cold Spring and Taylor/Upper Taylor Springs; Figures 1 and 2).

### Morphology

**Figure 4:**
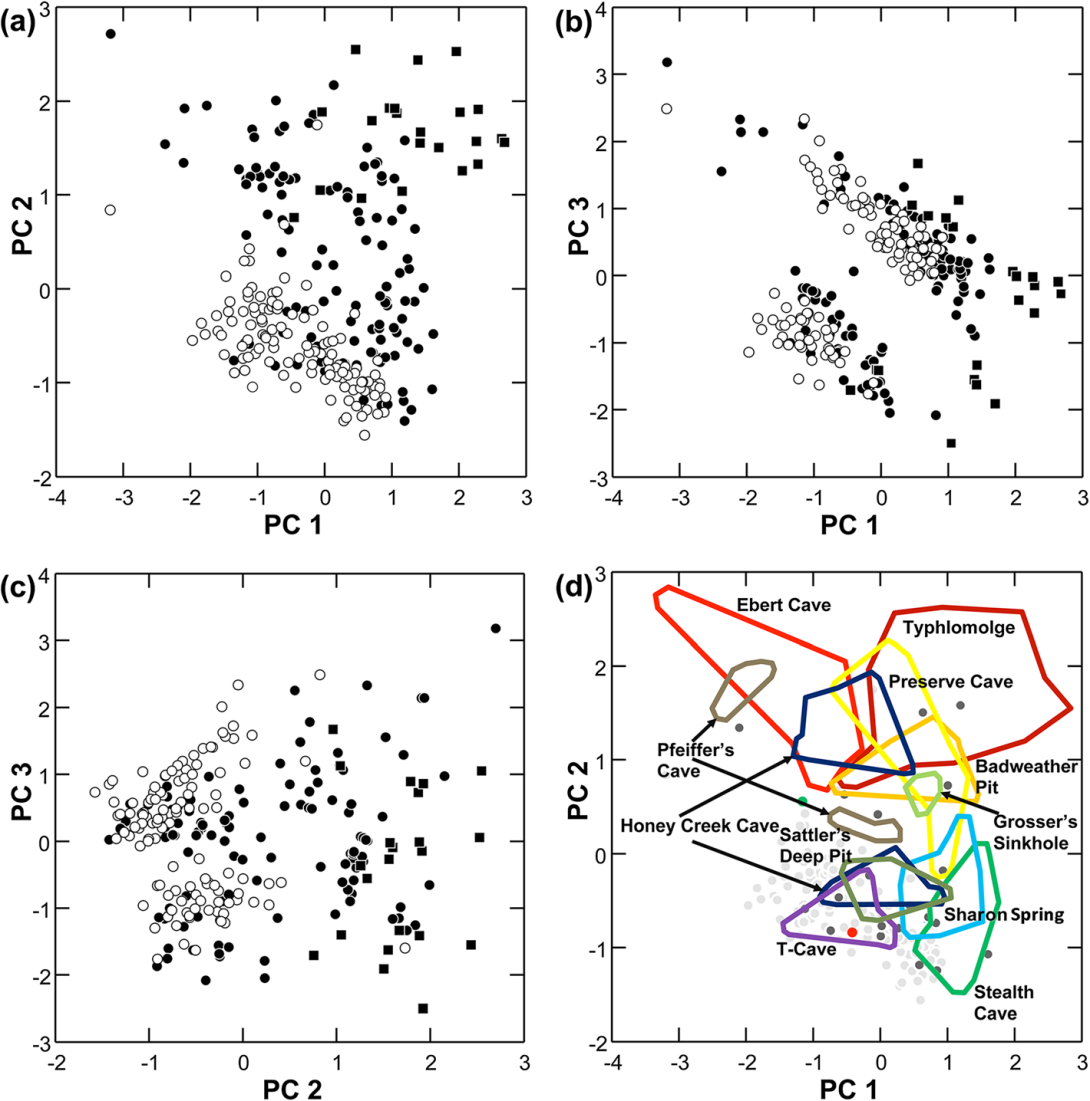
**Scatterplots of factor scores from principal components analysis (PCA) of log**_**10**_**-transformed measurements. 4a–c**: Ordination of specimens of the eastern *Blepsimolge* (circles) and *Typhlomolge* (squares; includes *E. rathbuni* and *E. waterlooensis*) clades are shown. Closed and open symbols represent specimens collected from cave and surface localities, respectively. **4d**: Ordination of morphological variation within and among cave populations of central Texas *Eurycea* (PC1 vs. PC2). Populations with *N* > 3 are shown with colored convex hull polygons (individual specimens removed except for outliers). Light gray circles indicate surface specimens; dark gray circles indicate cave specimens from localities with *N* ≤ 3.

The first three principal components (PCs) account for 96.9% of the total variance in the morphological data set. The first PC (78.7% of total variance) has high positive factor loadings for all variables, although somewhat lower for ED, and reflects the overall positive correlations between individual measurements and body size (Table 2). The second PC (9.5% of total variance) is structured primarily by the broad and diffuse variation exhibited by cave populations (Figure 4a). Along this axis, surface populations form a cohesive cluster with primarily negative factor scores. The most extreme troglomorphs (i.e., *Typhlomolge*) group together with high factor scores on both PC1 and PC2. Subterranean populations of the eastern *Blepsimolge*, particularly those assigned to *E. tridentifera*, overlap partially with *Typhlomolge*, but on average have slightly lower factor scores along PC1 and PC2. Thus, the combination of the first and second PCs, and particularly PC2, corresponds to a gradient from surface to cave morphologies, with surface specimens overlapping broadly in ordination space with cave specimens but not vice versa. PC2 was structured primarily by the inverse relationship between two sets of variables: AG and ED had high negative factor loadings while both HLL and IOD had high positive factor loadings. In summary, cave populations were characterized by small eye diameters and short axilla-groin lengths, and by long hind limbs and interocular distances.

**Table 2:** Factor loadings for variables, eigenvalues, and percent of total variance explained for principal components

**Measurement**	**PC 1**	**PC 2**	**PC 3**
AG	0.741	−0.315	−0.583
ALL	0.965	0.150	0.065
ED	0.342	−0.857	0.384
HLA	0.978	−0.006	0.128
HLB	0.970	0.101	0.149
HLC	0.979	0.032	0.157
HLL	0.960	0.174	0.100
HW	0.963	0.067	0.093
IOD	0.945	0.158	0.076
SL	0.821	−0.150	−0.538
**Eigenvalue**	7.870	0.949	0.868
**% Variance Explained**	78.7	9.5	8.7

Principal components were derived from the correlation matrix of log_10_-transformed morphometric data.

Morphological variation with respect to cave populations is complex (Figure 4). Some cave populations (e.g., Honey Creek Cave, Pfeiffer’s Cave) separate into at least two discrete groups in ordination space. For Honey Creek Cave (the type locality of *E. tridentifera*), one group is extremely troglomorphic while the other seems to be intermediate between troglomorphic and surface forms. For Pfeiffer’s Cave (near the type locality of *E. latitans*), both groups are well separated from each other, but also from the main cluster of surface forms. Only five cave populations (Preserve Cave, Honey Creek Cave, Badweather Pit, Camp Bullis Cave #1, and Camp Bullis Cave #3) overlapped partially in ordination space with the *Typhlomolge* specimens; thus, cave populations of eastern *Blepsimolge* were different from *Typhlomolge* in at least some aspects of morphology. Some of these populations exhibit distinctive troglomorphic variation, including Ebert Cave, Pfeiffer’s Cave, and Grosser’s Sinkhole. Yet other cave populations form relatively homogeneous clusters that were intermediate in ordination space between surface and extremely troglomorphic populations (e.g., Stealth Cave, Sharon Spring, and Sattler’s Deep Pit). Finally, specimens from T-Cave form a cohesive cluster indistinguishable from the main group of surface specimens.

**Figure 5:**
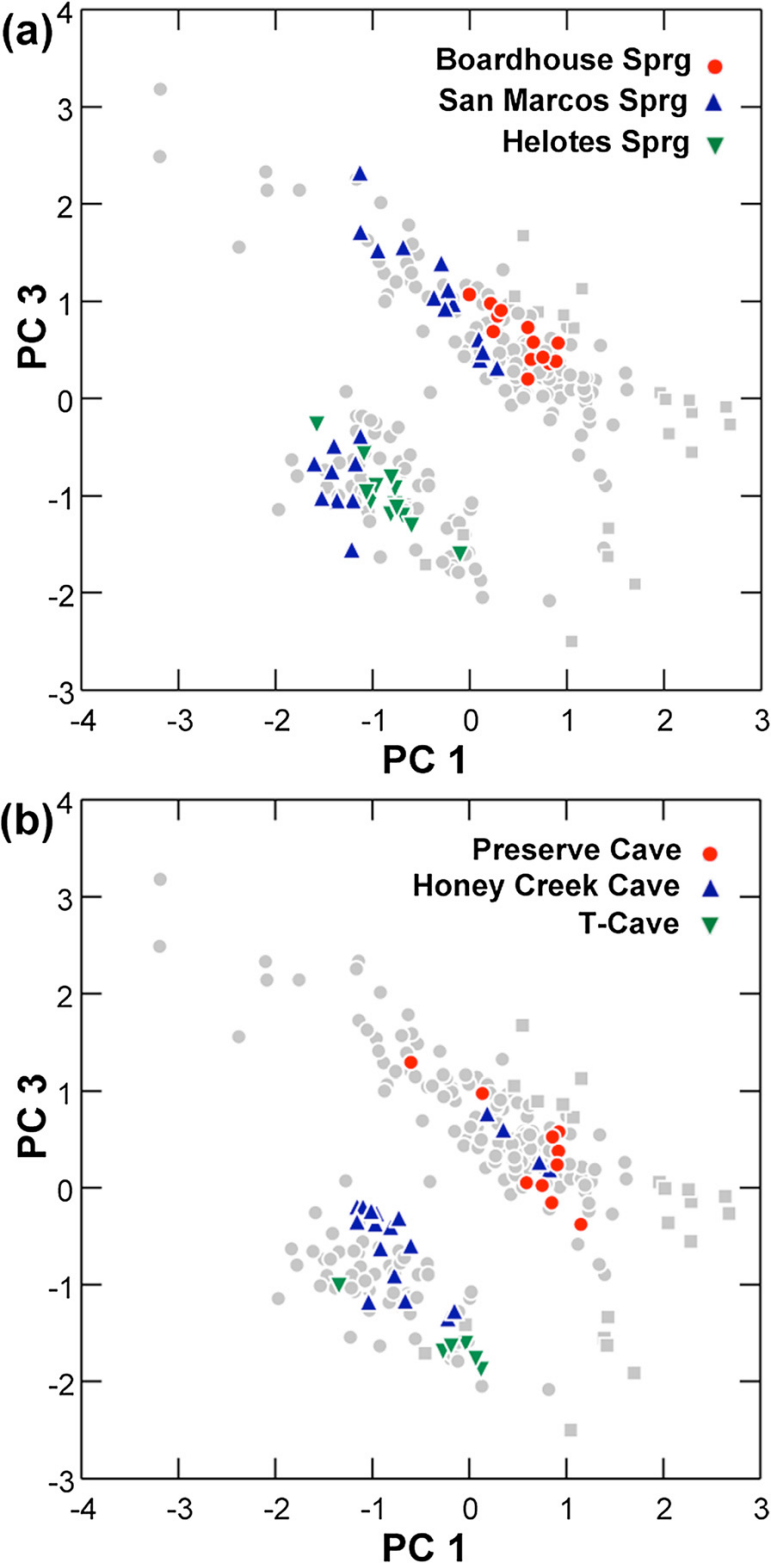
**Congruent patterns of morphological variation for selected surface (a) and cave (b) populations.** Localities are shown where specimens cluster in either of two groups or where specimens cluster in both groups. Light gray circles and squares indicate *Blepsimolge* and *Typhlomolge*, respectively.

The ordination of PC1 and PC3 (8.7% of total variance) reveals an unexpected and novel pattern of morphological variation in central Texas *Eurycea*: the eastern *Blepsimolge* form two discrete groups, with specimens of *Typhlomolge* mostly peripheral to or separate from these groups (Figure 4b). These distinct groups within eastern *Blepsimolge* do not correspond to geography, recognized species limits, phylogeographic structure, or habitat (unless at a scale finer than the cave/surface dichotomy used in this study). At the level of individual localities, cave and surface sites exhibited parallel patterns; some surface and cave populations include specimens from only one morphological group, while other populations are composed of specimens from both groups (Figure 5). A scatterplot of factor scores from each group indicates a negative relationship between PC3 and PC1 for each group (Figure 4b), which suggests that allometric differences influence group ordination. Although these groups are not structured on the basis of habitat designation, the variables that most strongly influence PC3 include measurements that were important in the gradient of surface-to-cave forms identified from PC2. The highest positive factor loading is for ED and the only negative loadings are for AG and SL (Table 2). In contrast, both AG and ED weight negatively on PC2. Thus, PC3 represents primarily residual, uncorrelated variation in ED and AG, variables that otherwise show a strong positive correlation along the gradient of surface-to-cave morphologies.

## Discussion

### Phylogeography

The mtDNA-based phylogeny shows a complex pattern in which three species, *E.* sp. Pedernales, *E. nana* and *E. sosorum*, are successively sister to a clade (the *Eurycea neotenes* complex) that includes *E. latitans*, *E. neotenes*, *E. pterophila*, *E. tridentifera*, plus other previously unassigned populations. Short branch lengths and lack of reciprocally monophyletic relationships to distinguish species within the *Eurycea neotenes* complex (Figure 1) indicate recent divergences coupled with incomplete lineage sorting and recent or ongoing gene flow (thus, potential conspecificity). Relationships among populations of the *Eurycea neotenes* complex follow a pattern that appears to be determined more by geographic proximity than by habitat (spring vs. cave) or morphology (Figure 2), and this pattern is not entirely consistent with currently recognized species boundaries (Figure 1).

This complex phylogeographic pattern likely reflects the dynamic nature of karst aquifer systems inhabited by central Texas *Eurycea*. The dissolution of limestone strata alters water flow routes over time, generating new connections between formerly disjunct populations and severing others in the process. On a shorter time scale, varying climatic conditions (e.g., floods or droughts) can influence hydrogeologic pathways [42] and transiently facilitate or hinder gene flow across the karstic landscape. Although geographic proximity is generally a good predictor of relatedness within the eastern *Blepsimolge*, there are many exceptions to this pattern that reflect the complexity of gene flow among salamanders within these karst aquifers. Some populations of *Eurycea* appear to be locally isolated while others have maintained genetic and hydrogeological connectivity with other populations. For example, White Springs is a small outflow in the Blanco River drainage, and based on its location, would be expected to have close genetic affinity to other Blanco and Guadalupe River populations (e.g., clades BP1, BP2, BGP). However, salamanders from White Springs are substantially divergent in mtDNA sequence and this population forms a polytomy with FT and N, which collectively are sister to the remaining members of the *E. neotenes* complex (Figure 1). In addition, the White Springs population is distinguished by several unique nuclear sequence alleles (PC, unpublished data). In two other instances we found that populations from geographically close springs appeared distantly related. Bullis springs 9–83 and 9–29 are adjacent to many N populations but are weakly supported as sister to the rest of the *E. neotenes* complex; FT populations are a very short geographic distance from LT populations as well but do not fall within that group (Figure 2).

In contrast to the above examples of more distantly related, but geographically proximate populations, other populations within the *E. neotenes* complex that have shared or similar haplotypes occur across a relatively wide geographic range despite potential barriers to gene flow. Because populations of eastern *Blepsimolge* are restricted to karst-associated waters (wet caves, springs, spring-fed streams), rivers can act as barriers to gene flow [10,43]. However, the BGP clade is distributed across two river drainages (Blanco and Guadalupe), suggesting recent gene flow and hydrogeological connectivity between these regions despite modern riverine barriers. For example, the Preserve Cave population (in which individuals exhibit troglomorphism similar to that of *E. tridentifera*; Figure 3) is south of the Guadalupe River but shares an identical haplotype with Horsejump Spring, which is north of the Blanco River. While gene flow between these populations may not currently be ongoing, their shared mitochondrial haplotype suggests recent hydrogeological connections and/or dispersal across contemporary barriers.

Temporary hydrogeological connections may also result in introgression between distinct species. *Eurycea sosorum* and *E. nana* inhabit springs that are discharge points for large regions of the Edwards Aquifer (the Barton Springs and San Antonio segments) and populations of both species from the type localities are morphologically and genetically distinctive [10,41,44,45]. However, the presence of *E. nana*-like haplotypes (clade S/N) throughout Barton Springs segment populations including *E. sosorum* (at a frequency of 100% in geographically intermediate springs and caves, and approximately 30% at Barton Springs itself; NB and PC, unpublished data [41]) indicates recent mtDNA introgression between these species. This is unexpected, since the traditional view of Edwards Aquifer hydrogeology suggested a groundwater divide between the Barton Springs segment, which drains north to Barton Springs, and a San Antonio segment, which drains to San Marcos and other large springs to the south (Figure 2; [46,47]). Recent dye tracing studies are beginning to challenge this view [47], as groundwater almost anywhere within Hays County may flow either north to Barton Springs (Travis County) or south to San Marcos Springs depending on environmental conditions (e.g., drought [42]). This hydrogeologic pattern may explain the presence of *E. nana*-like haplotypes within the Barton Springs segment. This and several other examples of shared haplotypes between genetically distinct groups (between N and LT; BGP and BP1) suggest that many of these species are not reproductively isolated. Although we cannot rule out shared ancestral polymorphism and incomplete lineage sorting, the fact that most of these cases occur among populations in relatively close geographic proximity (with the potential for hydrologic connection, past or present) is suggestive of at least sporadic gene flow.

Ecological segregation has also facilitated diversification of the eastern *Blepsimolge* through repeated colonization of subterranean habitats. However, genetic divergence between spring and cave populations generally is low despite the morphological diversity of the group. Most cave populations within the *E. neotenes* complex are distributed throughout the Lower Glen Rose (LGR) limestone formation and the most extreme troglomorphic forms (e.g., *E. tridentifera*) occur within the southern LGR (Figure 2). The morphological distinctiveness of *E. tridentifera* prompted Sweet [22] to suggest that these caves are among the oldest in the plateau region, which would have allowed ample time for the evolution of cave-associated features. Cavern development in this region coincided with the erosion of the Upper Glen Rose (UGR) limestone formation, exposing the LGR to extensive karstification. This process has been dated to approximately 1.3 Ma to 990 ka BP [48], putting a theoretical bound on the earliest cave colonization by surface populations. While cave-dwelling troglomorphs may have already inhabited UGR caves and later colonized newly available habitat when the LGR was karstified, this scenario is less likely given that the much lower extent of karstification exhibited by the UGR in this region [48] suggests that (1) the extensive subterranean habitat created within the LGR was novel, and (2) the extremely low mtDNA divergences between subterranean and surface populations within the *E. neotenes* complex are concordant with a pattern of recent rather than old colonization. The patterns of karst aquifer evolution and the complex evolutionary history of eastern *Blepsimolge* pose a challenging phylogeographic puzzle that likely will only be solved with additional sampling and incorporation of rapidly evolving nuclear markers as well as a better understanding of regional hydrogeology.

### Morphology

The remarkable array of morphological diversity in eastern *Blepsimolge* (Figure 3) is related to the extent to which populations exploit surface versus cave habitats. In general, subterranean forms show, to varying degrees, loss of pigmentation, shortening of the trunk, flattening and broadening of the skull, lengthening of the limbs, and reduction (and sometimes loss of function) of eyes. PCA indicates that surface salamanders occupy a relatively tight cluster in morphospace, while subterranean salamanders show more diffuse variation along both PC1 and PC2 (Figure 4). Overall, the ordination suggests that there are various “cave-type” morphologies in contrast to a more cohesive “surface-type” morphology. This observation is consistent with the results of previous studies that have attempted to assess species diversity within central Texas *Eurycea* using primarily morphological data (for review, see [10,20]).

The pattern of reduced variation in morphology of surface-dwelling salamanders relative to cave-dwelling populations may result from stronger stabilizing selection, which tends to reduce phenotypic variation. An obvious difference in selection pressure between cave and surface habitats is predation; salamander predators such as fishes, aquatic insects and crayfishes are mostly absent from caves in central Texas, but can be abundant on the surface (personal observations). Consistent with this idea, the diffuse variation exhibited by cave-dwelling salamanders may result from the relaxation of selection for traits that are important on the surface, and in particular those traits critical for evading predation. Our morphological data indicate asymmetric migration between habitats because surface forms are more frequently found in caves than vice versa. This observation may reflect low survivorship of troglomorphic salamanders that are flushed to the surface and temporary migration of surface populations due to periodic drying of the surface habitat [49]. However, the frequent parallel (sensu [17], given the close relationships) evolution of troglomorphic traits (see below) suggests that directional selection may be operating on cave-associated morphologies. For example, eye development and maintenance may be metabolically costly, and therefore selected against in perpetually dark environments [4,50]. Similarly, eye degeneration may arise through pleiotropic enhancement of other sensory organs [51]. Moreover, mechanosensory detection may be enhanced by changes in head and body shape, for example to reduce swimming noise or support larger numbers of superficial neuromasts (e.g., in amblyopsid fishes [52]). Extensive variation in morphology across different cave systems may result from differences in the following: (1) selection regimes, (2) time since invasion into various cave systems, (3) spatial extents and connectivity of caves (which may influence the dependence of some population segments on cave habitats), (4) habitat stability, and (5) frequencies of genetic admixture with surface populations and associated cave populations.

Most of the major mitochondrial clades recovered from our phylogeographic analyses include both cave and surface populations (Figure 1). Furthermore, mtDNA-based divergences between cave and surface populations in the eastern *Blepsimolge* are consistently small. These patterns, along with the overall low genetic diversity within the *E. neotenes* complex, indicate that troglomorphism arose rapidly and independently in the various cave populations (*contra* previous views, e.g., [22] re: *E. tridentifera*; populations assigned to this species do not form a well-differentiated, monophyletic group based on mtDNA sequence data). When considering mtDNA data alone, we cannot rule out the possibility that troglomorphic traits are maintained despite on-going gene flow from surface populations. Overall, this scenario seems most likely given that the phylogeographic structure reflects dynamic hydrogeological systems that connect subterranean and surface habitats, and that dispersal of surface forms is largely dependent on subterranean corridors.

Perhaps the most intriguing pattern recovered from our analysis of morphological data is the existence of two discrete groups separating primarily along PC3, mostly in association with PC1, but to a lesser extent with PC2 (Figure 5). These groups do not correspond to phylogeographic structure, previously established taxonomy, geography, or whether populations inhabit subterranean or surface habitats. In fact, individual localities, whether they are cave or surface sites, have individuals that group with one or the other cluster, or in both discrete clusters (Figure 5). Overall, these patterns indicate that the two morphological groups recovered are not influenced by geographic proximity or population-level genetic divergence. The three variables that weigh most heavily on PC3 are AG, SL, and ED (only AG and SL have negative factor loadings for this component), traits associated with morphological differences between surface and cave forms (Table 2). However, because PC2 clearly represents a surface-to-cave morphology gradient, the variation along PC3 that is separating specimens must be residual uncorrelated variation in these traits. Thus, a major aspect of the variation encompassed by PC3 seems to include reduction in eye diameter without concomitant reduction in trunk length, or vice-versa. The more distinct separation of these two groups along PC1 rather than PC2 indicates that scaling relationships are also involved in creating the pattern of group separation.

At least two factors may result in segregation of morphological variation into groups that do not correspond to geographic variation or genetic divergence. First, sexual dimorphism could generate this pattern, and in most cases the sexes of specimens measured could not reliably be determined. However, given that sexual differences in morphometric variables usually diverge with ontogeny from a common starting point [53,54], morphological dispersion based on such differences should not be discontinuous. Another possibility is developmental plasticity, which we consider to be the most plausible explanation for these groupings based on the limited data available. Developmental plasticity may operate through two mechanisms: (1) a threshold response to environmental stimuli whereby a developmental switch produces alternative forms (i.e., a developmental polyphenism), or (2) somatic or developmental selection, whereby large numbers of variants are produced and some variants are selectively preserved while others are eliminated [55]. The most important trait structuring variation along PC3 is trunk length, as indicated by similar correlation structure of AG and SL variables (although eye diameter does contribute somewhat as well). Trunk length is correlated with number of vertebral elements, which is determined by periodic somite formation in embryogenesis due to a molecular oscillator (i.e., the “segmentation clock”) [56,57]. Thus, plasticity can modify vertebral numbers by randomly adjusting rate parameters of the segmentation clock [58]. However, whether variation in the number of trunk elements has contributed to group separation remains unknown.

Plasticity itself is subject to selection, and the degree of plasticity in a trait is predicted to correlate with the amount of environmental variation to which the trait responds [55]. This facet of plasticity is important because the distinction between cave and surface habitats as they relate to salamander populations in central Texas is artificial in many cases. Salamanders from surface populations may spend considerable time in subterranean refugia, particularly when periods of drought cause surface springs to go dry [49]. Many populations rely on spring systems that are spatially heterogeneous in total available habitat both above and below ground. Furthermore, surface populations may depend more on interstitial groundwater than the open water column, and ease of movement through interstitial cavities may be influenced by features such as trunk length. Overall, the extreme heterogeneity and structural complexity of karst habitats may select for plasticity in traits such as somitogenesis and eye development, and somatic selection of variants may result in the discordant morphological patterns observed at the local scale. Developmental plasticity is also consistent with apparently rapid shifts in morphology between surface and cave populations, and the maintenance of different phenotypes despite the homogenizing influence of periodic gene flow. We cannot rule out other environmental influences (e.g., clutch effects [59]), but here the morphological extremes appear highly correlated with primary use of cave versus surface habitat.

### Taxonomic implications

We do not wish to provide a formal taxonomic treatment here (and thus, refrain from proposing taxonomic changes), but we offer comments on current species designations within the *E. neotenes* complex. *Eurycea tridentifera* Mitchell and Reddell [14] exhibits the most extreme cave-associated morphological features, which have long served as the basis for its taxonomic recognition [10,14,18,22,60,61]. The prevailing view was that this species represented a single lineage that independently evolved cave-associated morphological traits similar to those of *E. rathbuni*[17]. Allozyme frequency data also weakly supported its distinctiveness*,* although there were no fixed differences (in the three populations examined; [10]). Our analysis of multiple populations with various degrees of troglomorphism (including many more individuals assigned to *E. tridentifera* and previously unassigned, nearby populations whose members exhibit *tridentifera*-like morphologies) challenges this view. Populations formally assigned to *E. tridentifera* occur within the LT clade and are not distinct from other cave and surface populations in that group according to mtDNA sequence data (Figure 1). Salamanders from Preserve Cave are morphologically similar to those assigned to *E. tridentifera* (Figure 5), yet fall within the BGP clade, while the single *tridentifera*-like specimen from Hector Hole is part of the N clade (Figures 1 and 3). *Eurycea tridentifera* appears to be composed of populations closely related to surface forms that have evolved extreme troglomorphism independently (Figures 1, 3 and 4), and may not warrant recognition as a distinct species. Chippindale et al. [10] resurrected *E. latitans* Smith and Potter [62] from synonymy under *E. neotenes*, but regarded it as a “catch-all” group of problematic taxonomic status. Our results support this view, showing extensive mitochondrial polyphyly for populations assigned to this species (Figure 1). Sweet [22] regarded occurrence of salamanders with morphologies "intermediate" between those of surface and cave forms at Cascade Caverns (the type locality for *E. latitans*) as evidence of hybridization between *E. tridentifera* and *E. neotenes*, but we find no indication of this based on mtDNA, nor was this supported by allozyme data [10]. Thus, the status of *E. latitans* as a distinct species also is highly questionable. Chippindale et al. [10] tentatively recognized *E. pterophila* Burger, Smith and Potter [63] (which had been synonymized under *E. neotenes* by Sweet [19]) on the basis of similar allozyme frequencies (but no diagnostic alleles) and a geographic distribution exclusive to the Blanco River drainage [10]. While our results do show genetic affinities among some populations within the broader Blanco River watershed (e.g., clade BP2), other Blanco populations are more closely related to those within the Guadalupe watershed (including Rebecca Creek Spring, previously assigned to *E. latitans*[10]). This observation is also consistent with a population genetic study that documented evidence of genetic isolation among several populations of *E. pterophila*[43]. Clades containing populations assigned to *E. pterophila* form a polytomy with the LT clade (Figure 1) and divergence between these groups is low. Finally, populations assigned to *E. neotenes* Bishop and Wright [40], plus other cave and surface members of the N clade, form a largely cohesive group geographically.

The *E. neotenes* complex exhibits discordance with previously delimited species boundaries, contains a morphologically-based species unsupported by mtDNA evidence (*E. tridentifera*) and includes potentially cryptic species (e.g., White Springs). Extremely low genetic divergence between subterranean and surface populations is evident, and morphological groups do not correspond to clear geographic or phylogenetic patterns. Although the morphological variability of this group may have resulted in over-split taxonomy, there has also been lack of recognition of genetically divergent but morphologically similar species of Texas *Eurycea* (most notably those with “surface” morphologies, e.g., *E. chisholmensis*, *E. naufragia*, *E. tonkawae*[10] and *E. sosorum*[45]). We recognize that consistent morphological differences between populations may also be indicative of genomic divergence and we cannot rule out the possibility that this divergence may have occurred faster than mtDNA lineage sorting. Additionally, incongruence between species trees, mtDNA and nuclear gene trees has been documented in numerous cases [64-66], highlighting the potential pitfalls of relying solely on mitochondrial sequence data for taxonomic assessment. However, our results are not inconsistent with nuclear data presented in previous studies [10,43].

### Cave colonization, adaptation and speciation

Speciation in cave faunas has often been explained by two contrasting models: the ‘climate-relict’ and the ‘adaptive shift’ hypotheses. The distinction between these two models lies in whether vicariance or divergent selection pressures drive speciation [67-69]. Under the climate-relict hypothesis, speciation occurs when surface and cave populations are separated after climatic changes result in prolonged geographic isolation [70]. In this scenario, surface populations are extirpated, gene flow to cave populations is eliminated, and populations speciate in allopatry. The ‘adaptive-shift’ hypothesis explains speciation as a result of ecological niche separation [68,69]. In this case, ancestral “surface” populations are not isolated geographically from populations exploiting the novel cave niche [68,71]. Instead, natural selection drives differentiation and eventually severs gene flow between the incipient surface and cave sibling species [69].

The occurrence of phylogenetically-nested troglomorphic populations of spring salamanders (genus *Gyrinophilus*) within the geographic ranges of more widespread surface forms has been invoked as evidence for speciation with gene flow, and thus, supportive of an adaptive-shift hypothesis [72]. Based on our results, the adaptive-shift hypothesis is a better explanation of diversification and cave invasion (but not necessarily speciation) in eastern *Blepsimolge* than the climate-relict hypothesis because of (1) low divergence between spring and cave populations and (2) genetically similar (or indistinct) cave and spring forms occurring in sympatry. But how are disparate surface and cave morphologies maintained in spite of ecological overlap and apparent gene flow?

**Figure 6:**
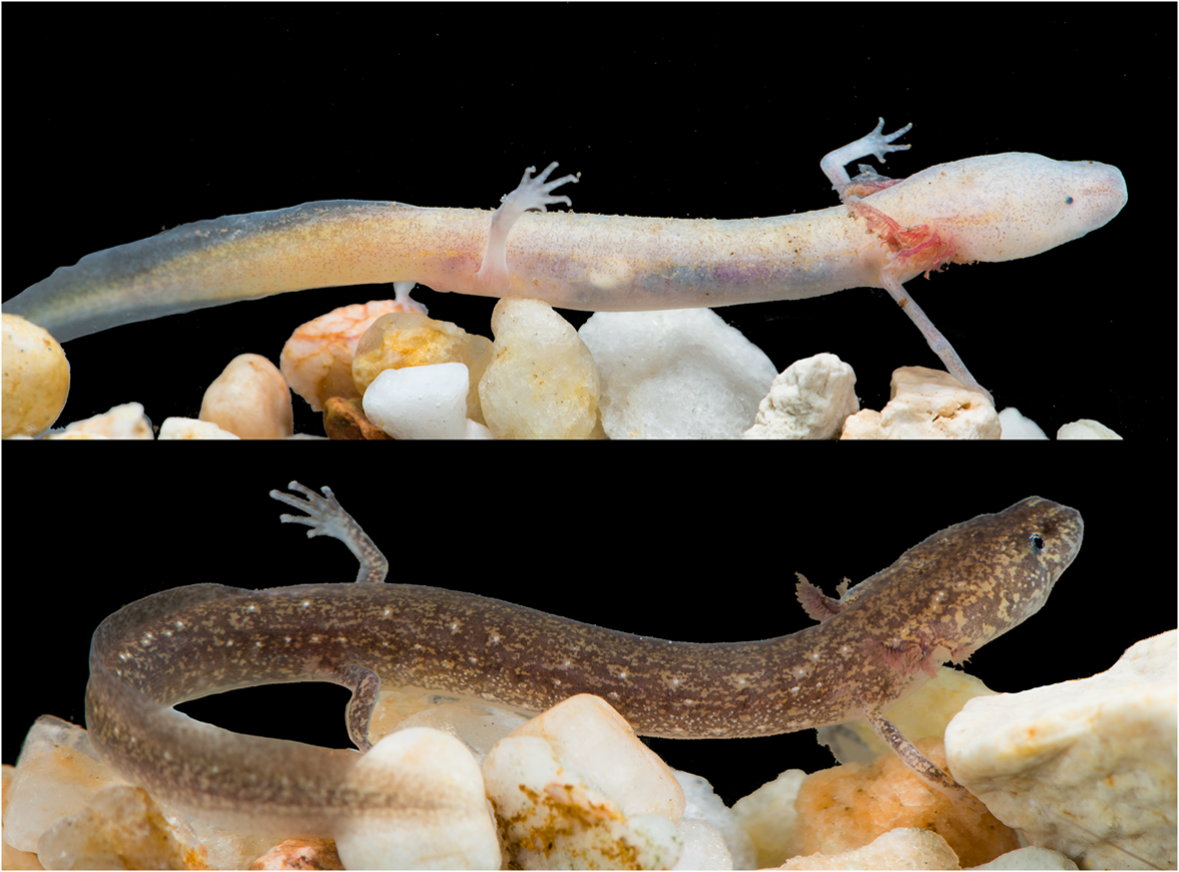
**Cave (top) and surface (bottom) morphs of *Eurycea *from Honey Creek Cave (type locality for *Eurycea tridentifera*).** These individuals were observed only meters apart within the same cave stream: the surface morph was encountered 5 m into the cave while the cave morph was observed approximately 20 m from the entrance.

Shared mtDNA haplotypes between surface and cave populations can result from incomplete lineage sorting, causing a lag between the process of lineage splitting and our ability to detect it [73]. However, there are several reasons why genetic admixture between surface and subterranean forms within the *E. neotenes* complex is likely. For example, populations of spring-dwelling central Texas *Eurycea* are dependent upon subterranean habitat [13], either for refuge from drought [49] or reproduction [74]. Additionally, several cave populations harbor a range of troglomorphic and surface forms (Figures 4d and 6) that share mitochondrial haplotypes. Thus, there is potential for extensive overlap between these niches, and our results suggest that sympatric surface and subterranean forms within the *E. neotenes* complex do not maintain isolation (although Sweet regarded this pattern as evidence for assortative mating among similar forms [22]). Whether troglomorphism arises during brief periods of isolation or arises in sympatry, the persistence of divergent morphological forms in genetically admixed populations may be due to strong selection for cave phenotypes [6], developmental plasticity, or both.

## Conclusions

The mt-gene phylogeny of eastern *Blepsimolge* reveals patterns of intermittent isolation and gene flow, a reflection of the dynamic nature of karst aquifers. Shallow genetic divergences among several recognized species suggest that the *E. neotenes* complex may have been over-split by early workers due to an emphasis on phenotypic divergence, particularly between cave and surface populations. This is in contrast to considerable cryptic species diversity among spring-dwelling populations of central Texas *Eurycea* owing to morphological conservatism among spring-dwellers. Evidence of genetic exchange and nested relationships across morphologically disparate cave and spring forms within the *E. neotenes* complex suggests that cave invasion in this group was recent and many troglomorphic morphologies (of individuals typically assigned to *E. tridentifera*) arose independently. These patterns are consistent with an adaptive-shift hypothesis of diversification. In many cases cave and surface forms do not appear to be genetically isolated, and even occur in microsympatry (Figure 6), suggesting that troglomorphism is maintained by strong selection and/or developmental plasticity.

### Availability of supporting data

Nucleotide sequence data supporting the results of this article are available in Genbank. Accession numbers are KC355860–KC355971 and KC355972–KC356083 for Cytb and ND2, respectively [see Additional file 1].

## Competing interests

The authors state that they have no competing interests for this publication.

## Authors’ contributions

NB, PC, and AG conceived the project; NB, PC, and AG conducted fieldwork and obtained tissue samples; NB and PC gathered molecular data; NB and JM performed statistical analyses; NB, JM, and PC wrote the manuscript; NB and JM prepared figures; AG collated information on hydrogeology and facilitated permits; CR obtained morphometric data; PC provided support and laboratory facilities; all authors edited and approved the final manuscript.

## Supplementary Material

Additional file 1 MS Excel file of collection and Genbank information for specimens used in genetic analysis. Click here for file

Additional file 2 MS Excel file of collection information for specimens used in morphometric analysis. Click here for file

## References

[B1] Wilkens H Strecker U Convergent evolution of the cavefish *Astyanax* (Characidae, Teleostei): genetic evidence from reduced eye‒size and pigmentation Biol J Linn Soc Lond 2003 80 545 554 10.1111/j.1095-8312.2003.00230.x

[B2] Xiao H Chen S Liu Z Zhang R Li W Zan R Zhang Y Molecular phylogeny of *Sinocyclocheilus* (Cypriniformes: Cyprinidae) inferred from mitochondrial DNA sequences Mol Phylogenet Evol 2005 36 67 77 10.1016/j.ympev.2004.12.007 15904857

[B3] Derkarabetian S Steinmann DB Hedin M Repeated and time-correlated morphological convergence in cave-dwelling harvestmen (Opiliones, Laniatores) from montane western North America PLoS ONE 2010 5 e10388 10.1371/journal.pone.0010388 20479884 PMC2866537

[B4] Culver DC Kane TC Fong DW Adaptation and Natural Selection in Caves: The Evolution of Gammarus minus 1995 Cambridge: Harvard University Press

[B5] Strecker U Bernatchez L Wilkens H Genetic divergence between cave and surface populations of *Astyanax* in Mexico (Characidae, Teleostei) Mol Ecol 2003 12 699 710 10.1046/j.1365-294X.2003.01753.x 12675825

[B6] Bradic M Beerli P León FJG Esquivel-Bobadilla S Borowsky RL Gene flow and population structure in the Mexican blind cavefish complex (*Astyanax mexicanus*) BMC Evol Biol 2012 12 9 10.1186/1471-2148-12-9 22269119 PMC3282648

[B7] Paquin P Hedin M The power and perils of “molecular taxonomy”: a case study of eyeless and endangered *Cicurina* (Araneae: Dictynidae) from Texas caves Mol Ecol 2004 13 3239 3255 10.1111/j.1365-294X.2004.02296.x 15367136

[B8] Juan C Guzik MT Jaume D Cooper SJB Evolution in caves: Darwin’s “wrecks of ancient life” in the molecular era Mol Ecol 2010 19 3865 3880 10.1111/j.1365-294X.2010.04759.x 20637049

[B9] Reddell JR The cave fauna of Texas with special reference to the western Edwards Plateau The Caves and Karst of Texas. A Guidebook for the 1994 Convention of the National Speleological Society with Emphasis on the Southwestern Edwards Plateau 1994 Huntsville: National Speleological Society 31 50

[B10] Chippindale PT Price AH Wiens JJ Hillis DM Phylogenetic relationships and systematic revision of central Texas hemidactyliine plethodontid salamanders Herpetol Monogr 2000 14 1 80

[B11] Potter FE Sweet SS Generic boundaries in Texas cave salamanders, and a redescription of *Typhlomolge robusta* (Amphibia: Plethodontidae) Copeia 1981 1981 64 75 10.2307/1444041

[B12] Sweet SS Natural metamorphosis in *Eurycea neotenes*, and the generic allocation of the Texas *Eurycea* (Amphibia: Plethodontidae) Herpetologica 1977 33 364 375

[B13] Sweet SS A distributional analysis of epigean populations of *Eurycea neotenes* in central Texas, with comments on the origin of troglobitic populations Herpetologica 1982 38 430 444

[B14] Mitchell RW Reddell JR *Eurycea tridentifera*, a new species of troglobitic salamander from Texas and a reclassification of *Typhlomolge rathbuni* Tex J Sci 1965 17 12 27

[B15] Bowles BD Sanders MS Hansen RS Ecology of the Jollyville Plateau salamander (*Eurycea tonkawae*: Plethodontidae) with an assessment of the potential effects of urbanization Hydrobiologia 2006 553 111 120 10.1007/s10750-005-5440-0

[B16] Chippindale PT Price AH Berkeley LM Conservation of Texas spring and cave salamanders (*Eurycea*) Amphibian Declines: The Conservation Status of United States Species 2005 Los Angeles: University of California Press 193 197

[B17] Wiens JJ Chippindale PT Hillis DM When are phylogenetic analyses misled by convergence? A case study in Texas cave salamanders Syst Biol 2003 52 501 514 12857641 10.1080/10635150390218222

[B18] Mitchell RW Smith RE Some aspects of the osteology and evolution of the neotenic spring and cave salamanders (*Eurycea*, Plethodontidae) of central Texas Tex J Sci 1972 23 343 362

[B19] Sweet SS On the status of *Eurycea pterophila* (Amphibia: Plethodontidae) Herpetologica 1978 34 101 108

[B20] Chippindale PT Bruce RC, Jaeger R, Houck LD Species boundaries and species diversity in the central Texas hemidactyliine plethodontid salamanders, genus *Eurycea* The Biology of Plethodontid Salamanders 2000 New York: Kluwer Academic/Plenum Publishers 149 165

[B21] Hillis DM Chamberlain DA Wilcox TP Chippindale PT A new species of subterranean blind salamander (Plethodontidae: Hemidactyliini: *Eurycea: Typhlomolge*) from Austin, Texas, and a systematic revision of central Texas paedomorphic salamanders Herpetologica 2001 57 266 280

[B22] Sweet SS Secondary contact and hybridization in the Texas cave salamanders *Eurycea neotenes* and *E. tridentifera* Copeia 1984 1984 428 441 10.2307/1445201

[B23] Cantino PD de Queiroz K Phylocodehttp://www.ohio.edu/phylocode

[B24] Wiens JJ Engstrom TN Chippindale PT Rapid diversification, incomplete isolation, and the “speciation clock” in North American salamanders (genus *Plethodon*): testing the hybrid swarm hypothesis of rapid radiation Evolution 2006 60 2585 2603 17263119

[B25] Hillis DM Mable BK Larson A Davis SK Zimmer EA Hillis DM, Moritz C, Mable BK Nucleic acids IV: sequencing and cloning Molecular Systematics 1996 2 Sunderland: Sinauer Associates 321 381

[B26] Walsh PS Metzger DA Higuchi R Chelex 100 as a medium for simple extraction of DNA for PCR-based typing from forensic material Biotechniques 1991 10 506 513 1867860

[B27] Moritz C Schneider CJ Wake DB Evolutionary relationships within the *Ensatina eschscholtzii* complex confirm the ring species interpretation Syst Biol 1992 41 273 291

[B28] Vences M Thomas M Bonett RM Vieites DR Deciphering amphibian diversity through DNA barcoding: chances and challenges Philos Trans R Soc Lond B Biol Sci 2005 360 1859 1868 10.1098/rstb.2005.1717 16221604 PMC1609216

[B29] Tamura K Peterson D Peterson N Stecher G Nei M Kumar S MEGA5: molecular evolutionary genetics analysis using maximum likelihood, evolutionary distance, and maximum parsimony methods Mol Biol Evol 2011 28 2731 2739 10.1093/molbev/msr121 21546353 PMC3203626

[B30] Edgar RC MUSCLE: multiple sequence alignment with high accuracy and high throughput Nucleic Acids Res 2004 32 1792 1797 10.1093/nar/gkh340 15034147 PMC390337

[B31] Ronquist F Huelsenbeck JP MrBayes 3: Bayesian phylogenetic inference under mixed models Bioinformatics 2003 19 1572 1574 10.1093/bioinformatics/btg180 12912839

[B32] Miller MA Pfeiffer W Schwartz T Creating the CIPRES Science Gateway for inference of large phylogenetic trees 2010 Gateway Computing Environments Workshop: 14 November 2010; New Orleans 2010 Washington DC, USA: IEEE 1 8

[B33] Guindon S Gascuel O A simple, fast, and accurate algorithm to estimate large phylogenies by maximum likelihood Syst Biol 2003 52 696 704 10.1080/10635150390235520 14530136

[B34] Posada D jModelTest: phylogenetic model averaging Mol Biol Evol 2008 25 1253 1256 10.1093/molbev/msn083 18397919

[B35] Marshall DC Cryptic failure of partitioned Bayesian phylogenetic analyses: lost in the land of long trees Syst Biol 2010 59 108 117 10.1093/sysbio/syp080 20525623

[B36] Rambaut A Drummond AJ Tracer v1.5 2009 http://tree.bio.ed.ac.uk/software/tracer/ [Accessed March 12 2013]

[B37] Marroig G When size makes a difference: allometry, life-history and morphological evolution of capuchins (*Cebus*) and squirrels (*Saimiri*) monkeys (Cebinae, Platyrrhini) BMC Evol Biol 2007 7 20 10.1186/1471-2148-7-20 17300728 PMC1808050

[B38] McCoy MW Bolker BM Osenberg CW Miner BG Vonesh JR Size correction: comparing morphological traits among populations and environments Oecologia 2006 148 547 554 10.1007/s00442-006-0403-6 16604370

[B39] Adams DC Rohlf FJ Slice DE Geometric morphometrics: ten years of progress following the “revolution. Italian Journal of Zoology 2004 71 5 16 10.1080/11250000409356545

[B40] Bishop SC Wright M A new neotenic salamander from Texas Proc Biol Soc Wash 1937 50 141 144

[B41] Chippindale PT Status of newly discovered cave and spring salamanders (Eurycea) in southern Travis and northern Hays Counties 2012 Austin, Texas: Texas Parks and Wildlife Department 31

[B42] Johnson S Schindel G Veni G Hauwert N Hunt B Smith B Gary M Tracing groundwater flowpaths in the vicinity of San Marcos Springs, Texas 2012 San Antonio, Texas: Edwards Aquifer Authority 139

[B43] Lucas L Gompert Z Ott J Nice C Geographic and genetic isolation in spring-associated *Eurycea* salamanders endemic to the Edwards Plateau region of Texas Conserv Genet 2009 10 1309 1319 10.1007/s10592-008-9710-2

[B44] Chippindale PT Price AH Hillis DM Systematic status of the San Marcos salamander, *Eurycea nana* (Caudata: Plethodontidae) Copeia 1998 1998 1046 1049 10.2307/1447356

[B45] Chippindale PT Price AH Hillis DM A new species of perennibranchiate salamander (*Eurycea*: Plethodontidae) from Austin, Texas Herpetologica 1993 49 248 259

[B46] Andrews F Schertz T Slade RJ Rawson J Effects of storm-water runoff on water quality of the Edwards Aquifer near Austin, Texas. Water-Resources Investigations Report 1984 Austin, Texa: United States Geological Survey 50

[B47] Hauwert NM Groundwater flow and recharge within the Barton Springs Segment of the Edwards Aquifer, southern Travis and northern Hays Counties, Texas PhD thesis 2009 The University of Texas at Austin: Department of Geological Sciences

[B48] Veni G Geomorphology, hydrology, geochemistry, and evolution of the karstic lower Glen Rose aquifer, south-central Texas PhD thesis 1994 The Pennsylvania State University: Department of Geosciences

[B49] Bendik NF Gluesenkamp AG Body length shrinkage in an endangered amphibian is associated with drought J Zool 2013 290 35 41 10.1111/jzo.12009

[B50] Protas M Conrad M Gross JB Tabin C Borowsky R Regressive evolution in the Mexican cave tetra, *Astyanax mexicanus* Curr Biol 2007 17 452 454 10.1016/j.cub.2007.01.051 17306543 PMC2570642

[B51] Yamamoto Y Byerly MS Jackman WR Jeffery WR Pleiotropic functions of embryonic sonic hedgehog expression link jaw and taste bud amplification with eye loss during cavefish evolution Dev Biol 2009 330 200 211 10.1016/j.ydbio.2009.03.003 19285488 PMC3592972

[B52] Niemiller ML Poulson TL Trajano E, Bichuette ME, Kapoor BG Subterranean fishes of North America: Amblyopsidae Biology of Subterranean Fishes 2010 Enfield: Science Publishers 169 280

[B53] Badyaev AV Growing apart: an ontogenetic perspective on the evolution of sexual size dimorphism Trends Ecol Evol 2002 17 369 378 10.1016/S0169-5347(02)02569-7

[B54] Gluesenkamp AG Acosta N Sexual dimorphism in *Osornophryne guacamayo* with notes on natural history and reproduction in the species J Herpetol 2001 35 148 151 10.2307/1566040

[B55] West-Eberhard MJ Developmental Plasticity and Evolution 2003 1 New York: Oxford University Press

[B56] Cooke J Zeeman EC A clock and wavefront model for control of the number of repeated structures during animal morphogenesis J Theor Biol 1976 58 455 476 10.1016/S0022-5193(76)80131-2 940335

[B57] Gomez C Özbudak EM Wunderlich J Baumann D Lewis J Pourquié O Control of segment number in vertebrate embryos Nature 2008 454 335 339 10.1038/nature07020 18563087

[B58] Müller J Scheyer TM Head JJ Barrett PM Werneburg I Ericson PGP Pol D Sánchez-Villagra MR Homeotic effects, somitogenesis and the evolution of vertebral numbers in recent and fossil amniotes Proc Natl Acad Sci USA 2010 107 2118 2123 10.1073/pnas.0912622107 20080660 PMC2836685

[B59] Adams DC Quantitative genetics and evolution of head shape in *Plethodon* salamanders Evol Biol 2011 38 278 286 10.1007/s11692-011-9120-0

[B60] Wake DB Comparative osteology and evolution of the lungless salamanders, family Plethodontidae Memoirs of the Southern California Academy of Sciences 1966 4 1 111

[B61] Sweet SS Eurycea tridentifera Catalogue of American Amphibians and Reptiles 1977 199 1 199

[B62] Smith HM Potter FE Jr A third neotenic salamander of the genus *Eurycea* from Texas Herpetologica 1946 3 105 109

[B63] Burger W Smith HM Floyd E Potter: Another neotenic *Eurycea *from the Edwards Plateau Proceedings of the Biological Society of Washington, D 1950 63 51 57

[B64] Shaw KL Conflict between nuclear and mitochondrial DNA phylogenies of a recent species radiation: what mtDNA reveals and conceals about modes of speciation in Hawaiian crickets Proc Natl Acad Sci USA 2002 99 16122 16127 10.1073/pnas.242585899 12451181 PMC138575

[B65] Fisher-Reid MC Wiens JJ What are the consequences of combining nuclear and mitochondrial data for phylogenetic analysis? Lessons from *Plethodon* salamanders and 13 other vertebrate clades BMC Evol Biol 2011 11 300 10.1186/1471-2148-11-300 21995558 PMC3203092

[B66] Leaché AD Species trees for spiny lizards (genus *Sceloporus*): identifying points of concordance and conflict between nuclear and mitochondrial data Mol Phylogenet Evol 2010 54 162 171 10.1016/j.ympev.2009.09.006 19751840

[B67] Desutter-Grandcolas L Grandcolas P The evolution toward troglobitic life: a phylogenetic reappraisal of climatic relict and local habitat shift hypotheses Mémoires de Biospléologie 1996 23 57 6324058932

[B68] Howarth FG The evolution of non-relictual tropical troglobites Int J Speleol 1987 16 1 16 10.5038/1827-806X.16.1.1

[B69] Rivera MAJ Howarth FG Taiti S Roderick GK Evolution in Hawaiian cave-adapted isopods (Oniscidea: Philosciidae): vicariant speciation or adaptive shifts? Mol Phylogenet Evol 2002 25 1 9 10.1016/S1055-7903(02)00353-6 12383746

[B70] Peck, Finston TL Galapagos Islands troglobites: the questions of tropical troglobites, parapatric distributions with eyed-sister-species, and their origin by parapatric speciation Mémoires de Biospléologie 1993 20 19 3724058932

[B71] Howarth F High-stress subterranean habitats and evolutionary change in cave-inhabiting arthropods Am Nat 1993 142 S65 S77 10.1086/285523 19425953

[B72] Niemiller ML Fitzpatrick BM Miller BT Recent divergence with gene flow in Tennessee cave salamanders (Plethodontidae: *Gyrinophilus*) inferred from gene genealogies Mol Ecol 2008 17 2258 2275 10.1111/j.1365-294X.2008.03750.x 18410292

[B73] Wiens JJ Penkrot TA Delimiting species using DNA and morphological variation and discordant species limits in spiny lizards (*Sceloporus*) Syst Biol 2002 51 69 91 10.1080/106351502753475880 11943093

[B74] City of Austin Biological Assessment Barton Springs Flood Debris Removal and Bypass Repairs, Austin, Texas 2010 Austin, Texas: U.S. Army Corps of Engineers, Ft. Worth District Office

